# From *p*-values to Bayes Factor: A Meta-Analytic Comparison in Colorectal Research

**DOI:** 10.1007/s13193-025-02314-8

**Published:** 2025-04-25

**Authors:** Mufaddal Kazi

**Affiliations:** 1 Department of Surgical Oncology, Tata Memorial Hospital and Advanced Centre for Treatment Research, and Education, Homi Bhabha National Institute, Navi Mumbai, 410210 India; 2 Division of Colorectal Surgery, Department of Surgical Oncology, Tata Memorial Hospital, Mumbai, 400012 India; 3 Department of Surgery, Homi Bhabha National Institute, Mumbai, India

**Keywords:** Meta-analysis, Bayesian, Colorectal, Bayes factor, Indocyanine green, Trans-anastomotic tubes

## Abstract

The prevalent method for synthesizing evidence from multiple studies is the frequentist meta-analysis, which relies on assumptions of long-term frequencies and does not directly address the probability of hypotheses. In contrast, the Bayesian meta-analysis provides a framework that integrates prior knowledge with observed data, offering a more nuanced interpretation. This study aims to compare the outputs and interpretations of frequentist and Bayesian meta-analyses using published trials on colorectal anastomosis as examples. Two previously published meta-analyses on colorectal anastomosis—one evaluating trans-anastomotic tubes (TAT) and the other indocyanine green (ICG) fluorescence imaging—were reanalysed using frequentist and Bayesian approaches. Sequential Bayesian analyses were also conducted, updating priors with each additional study. Results were presented using odds ratios (OR), confidence intervals (CI), credible intervals (CrI), *p*-values, and Bayes factors (BF_10_). Both methods produced nearly similar ORs for the TAT meta-analysis; however, the Bayesian approach yielded slightly narrower CrIs and a BF_10_ that indicated a slight preference for the null hypothesis that was unclear with *p*-values alone. In the ICG meta-analysis, the Bayesian analysis produced a BF_10_ suggesting that it was 19 times more likely to observe the data under the assumption that the alternative hypothesis is true compared to the null, considerably making the estimates more conservative than the frequentist output. The Bayesian sequential analysis demonstrated increasing confidence in the alternate hypothesis with the addition of more studies. While frequentist and Bayesian meta-analyses may produce similar point estimates based on prior evidence, their interpretations and implications for hypothesis testing differ significantly. Bayesian methods offer a more flexible and intuitive approach, particularly in contexts with prior knowledge or when sequential updating is required. While frequentist outputs depend on multiple experiments, assuming that the null is true, and heavy dependence on conventional *p*-value thresholds, Bayesian outputs provide the direct probability of the hypothesis in question and credible intervals that are likely to contain the true estimate.

## Introduction

Frequently in practice, multiple trials on the same topic have dissimilar results. The differences may be in the magnitude of estimates, their direction, intervals, and the assumed statistical significance (*p*-value thresholds). How should trial results be interpreted to inform practice? The problem is often compounded when trials do not achieve the set statistical thresholds and have to be reported as negative based on conventional assumptions. Pragmatic and logistic considerations regularly influence the investigators to overestimate the benefit to reduce the sample size, consequently making them underpowered to detect clinically meaningful benefits. When several similar studies report negative results, it compels the readers to believe that no true difference exists.

However, that is far from the truth, and a more mathematical and unbiased perspective is needed. The popular method is the conventional (frequentist) meta-analysis when trials report on the same subject with a nearly similar methodology. There are two other ways of combining results, namely, Bayesian meta-analysis and its modifications, and the cumulative or sequential updating of trials. The focus of further discussion will largely be on the latter two methods in comparison with the frequentist (conventional) meta-analytic interpretation. A brief introduction to Bayesian methodology is necessary for subsequent deliberations. Bayesian philosophy is that of conditional probability and measures the chance of an event occurring given another event is known; for our purposes, the probability of the hypothesis given the observed data. The heart of Bayesian inference is that the updated beliefs (posterior) are based on data (likelihood) and the initial belief (prior). The posterior odds are simply the product of the likelihood ratio and the prior odds [1].

The difference in the outputs of the two statistical computation methods is fundamental. The frequentist statistics assumes that the null hypothesis is true, relies on multiple experimentations, and provides no direct insights about the tested alternate hypothesis. In conditional probability terms, a *p*-value is the probability of observing the given data given that the null hypothesis was true. At the same time, the true value (population mean) would be contained in 95% of the intervals generated if the experimentation was carried out an infinite number of times. Contrast this to Bayesian outputs of credible intervals (CrI), which directly provide the probability of having the true value within the interval provided from a single experiment incorporating prior beliefs. Similarly, the Bayes factor is precisely the ratio of the probability of observing the data provided that the alternate hypothesis is true over the null.

The subsequent analysis aims to compare the outputs of different methods of combining study results and to contrast the interpretations from frequentist and Bayesian viewpoints using published meta-analyses on colorectal anastomosis. Readers may choose to go directly to the discussion section, avoiding the methods and results, for a narrative description and differential implications of the two analytic methods.

## Methods

Trials on two colorectal topics were chosen from recently published meta-analyses of randomized controlled trials (RCT) alone for this discussion. One meta-analysis reported on the use of trans-anastomotic tubes (TAT) and the other on the use of indocyanine green (ICG) for the prevention of anastomotic leaks [2, 3]. Both of these meta-analyses used frequentist approaches, using the random-effects (RE) model alone and reported on anastomotic leaks as their primary outcome. The individual log odds ratio of the included studies in the meta-analysis was extracted, along with the confidence intervals to derive the standard errors.

A Bayesian meta-analysis was performed using the extracted data on the same trials, using non-informative or flat priors for the outcome estimate and heterogeneity. The distributions for the outcome were set for Cauchy (0, 0.707) and heterogeneity as inverse gamma (1, 0.15). A Bayesian model–averaged meta-analysis for fixed- and random-effect was summarized. The forest plots of the log odds ratio for the Bayesian method provide the observed and estimated effect sizes for each study. In addition, the summary estimates demonstrate the outcomes with fixed-effects (FE), RE, and for the model-averaged for the FE and RE. Outcomes were reported as mean estimates of the log odds ratio, 95% credible intervals, and the model-averaged Bayes factor (BF_10_) for the alternative hypothesis (H_1_) over the null hypothesis (H_0_). Back-transformation of the log odds ratio to odds ratio (OR) was performed.

Next, sequential analyses of trials were performed based on the chronological order of publication. Starting with a null prior with the above-described distributions, trials were added sequentially, and cumulative outcomes were assessed, which became the prior for the next trial addition. Results were presented in cumulative forest plots and graphs for sequential BF_10_.

Analyses were performed using R with the metafor package for a conventional frequentist meta-analysis and the metaBMA package for the Bayesian model–averaged meta-analysis [4, 5].

## Results

**Fig. 1:**
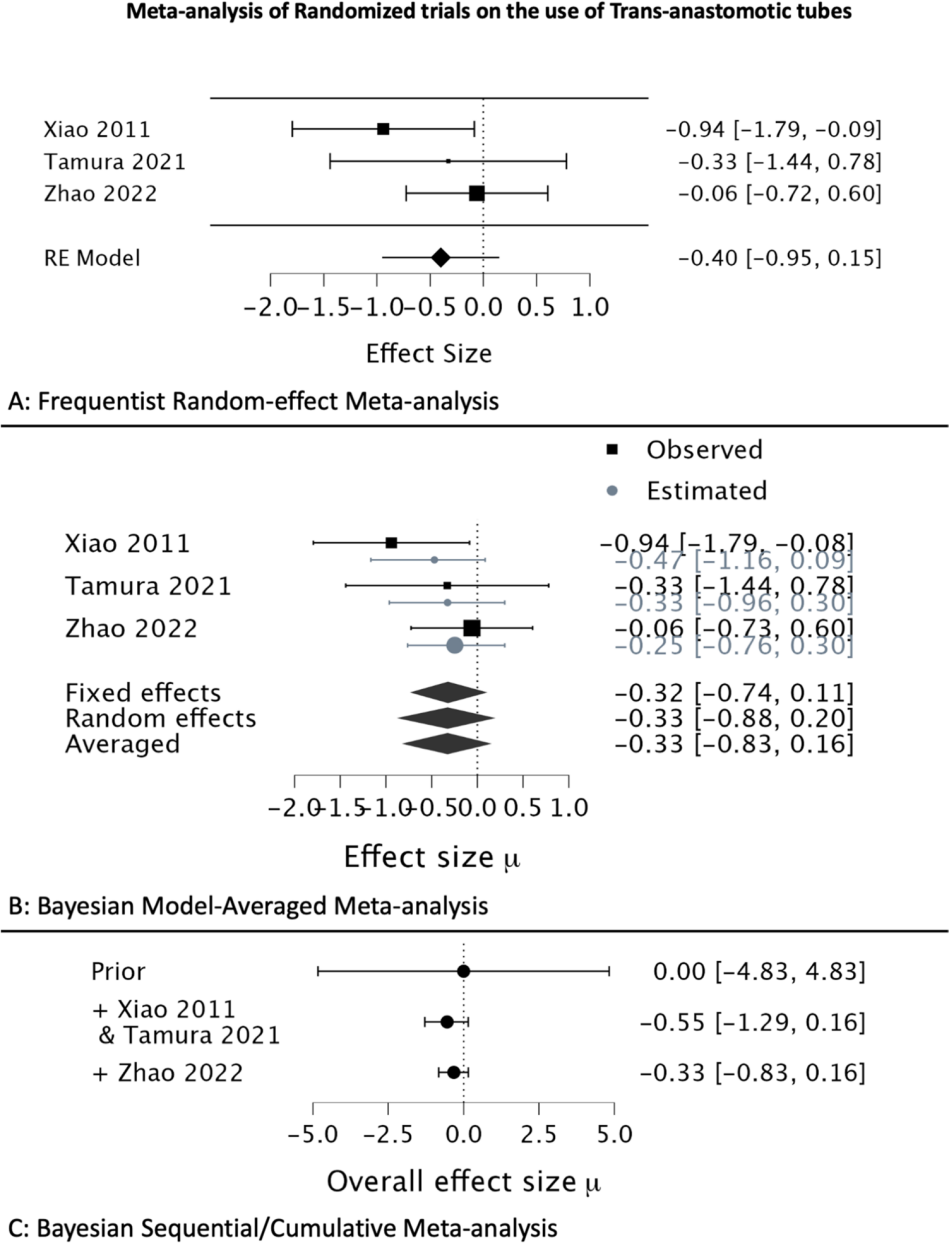
Meta-analysis of randomized trials on the use of trans-anastomotic tubes. **A** Frequentist Random-effect meta-analysis; **B** Bayesian model–averaged meta-analysis; **C** Bayesian sequential/cumulative meta-analysis

**Fig. 2:**
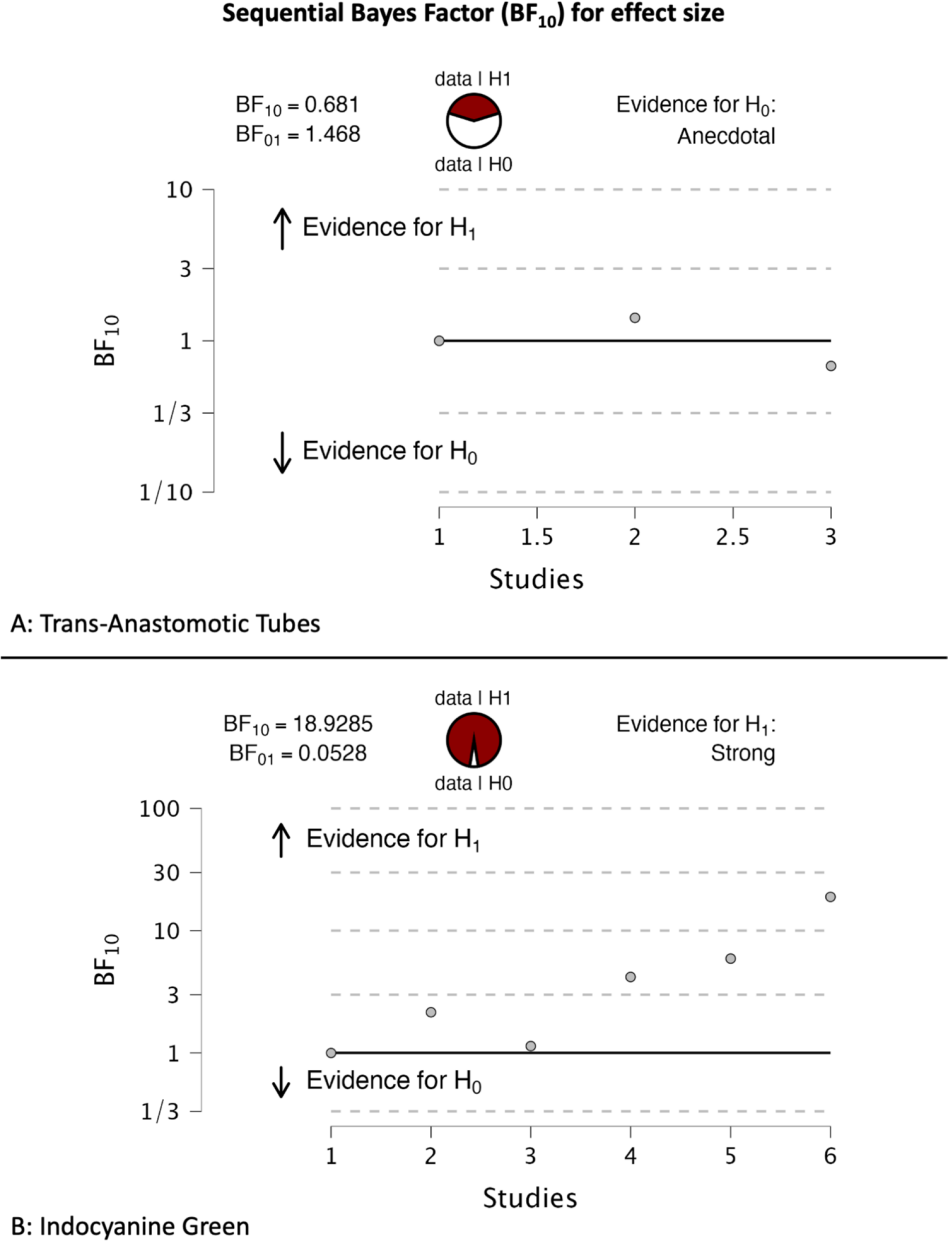
Sequential Bayes factor (BF_10_) for effect size. **A** Trans-anastomotic tubes; **B** indocyanine green

The meta-analysis on the use of TAT had three RCTs [6–8], and the conventional meta-analysis resulted in an OR of 0.670 with a 95% CI of 0.386 to 1.162, giving a *p*-value of 0.15 (Table 1). The Bayesian model–averaged methods resulted in an OR of 0.719 and 95% CrI of 0.43 to 1.17, providing a BF_10_ of 0.681. Both methods resulted in similar point estimates and intervals favoring the use of TAT. However, neither the *p*-values nor the BF_10_ provided sufficient evidence against H_0_. The forest plots of the Bayesian methods demonstrate that the estimated effects for individual studies appear to shrink towards the average (Fig. 1). Finally, a cumulative forest plot demonstrates the change in estimates and intervals with the sequential addition of studies beginning with a null prior, and the sequential BF_10_ plot demonstrates how the BF_10_ changes cumulating studies (Fig. 2). A BF_10_ of 0.681 suggests that the H_0_ is 1.47 times more likely than the H_1_.

**Table 1:** Output of estimates using frequentist and Bayesian methods for two meta-analyses

		Trans-anastomotic tubes	Indocyanine green
Frequentist (random-effects)	Log odds ratio	− 0.40 (− 0.95, 0.15)	− 0.53 (− 0.83, − 0.23)
	Heterogeneity	Tau 0.0522; *I*^2^ 21.42%	Tau 0; *I*^2^ 0%
	Estimate (odds ratio)	0.67	0.625
	95% confidence intervals	0.386–1.162	0.437–0.894
	*p*-values	0.15	0.0005
Bayesian (model-average)	Log odds ratio	− 0.33 (− 0.83,0.16)	− 0.50 (− 0.82, − 0.18)
	Estimate (odds ratio)	0.719	0.607
	95% credible intervals	0.43–1.17	0.440–0.835
	Bayes factor (BF_10_)	0.681	18.928

**Fig. 3:**
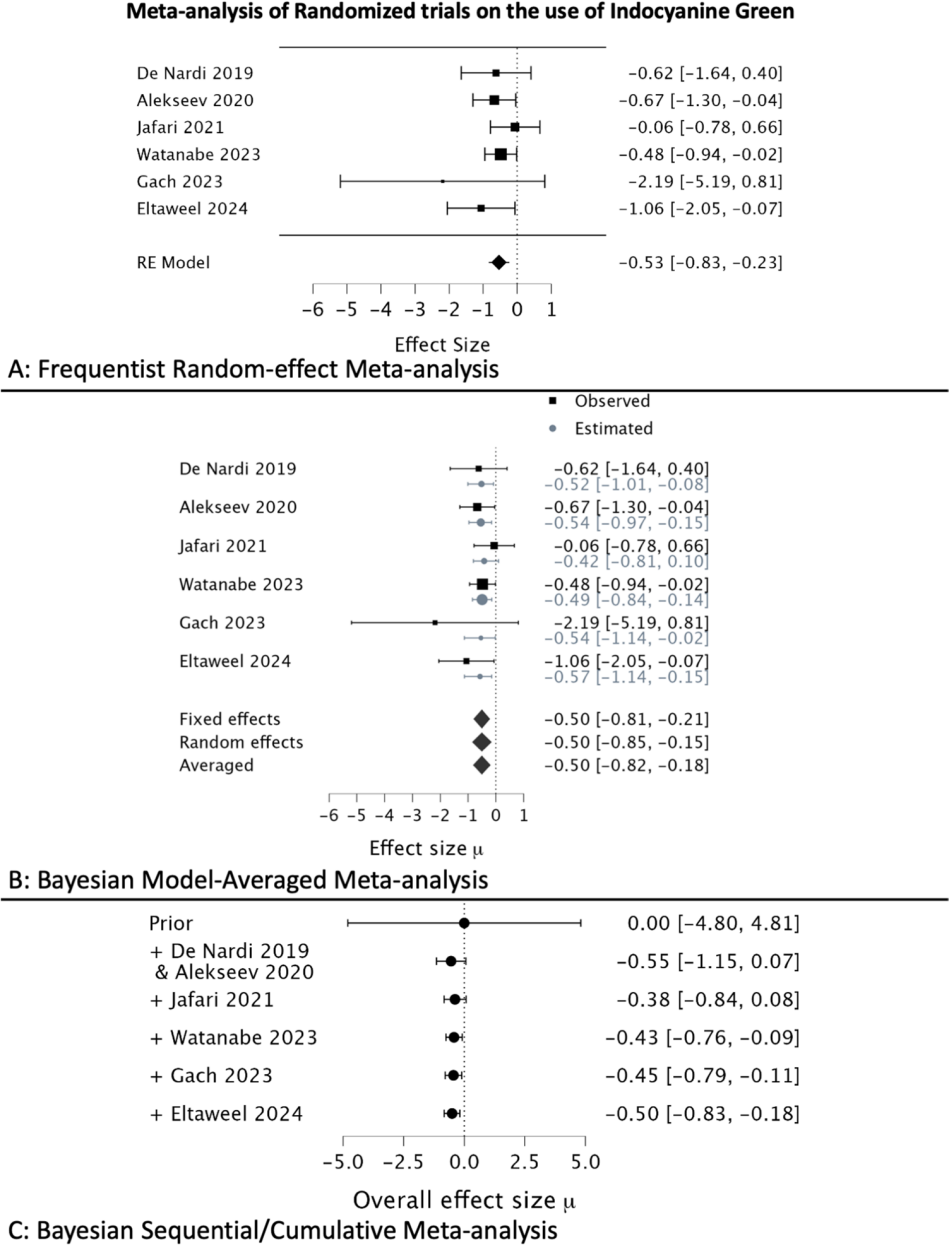
Meta-analysis of randomized trials on the use of Indocyanine green. **A** Frequentist random-effect meta-analysis; **B** Bayesian model–averaged meta-analysis; **C** Bayesian sequential/cumulative meta-analysis

The second meta-analysis on the use of ICG fluorescence perfusion imaging summarized six RCTs [9–14]. The frequentist estimates were an OR of 0.625 and a 95% CI of 0.437 to 0.894 (*p*− 0.0005), supporting the use of ICG in reducing anastomotic leaks (Table 1). Again, the Bayesian estimated OR was 0.607 with a 95% CrI of 0.440 to 0.835. The BF_10_ was 18.93, suggesting the probability of observing the given data was 19 times more likely under the assumption that H_1_ was true over H_0_. Despite the considerable variation in individual study effect sizes, the estimated effect sizes from Bayesian calculations fluctuated substantially less, and the same was detected in the cumulative analysis (Fig. 3). The BF_10_ shows a sequential increase, demonstrating an increasing probability for H_1_ with the addition of studies without changing the estimated effect size.

## Discussion

Two published meta-analyses on interventions to reduce colorectal anastomotic leaks were re-analyzed using the Bayesian model–averaged and sequential Bayesian methods, and their outputs were compared with the conventional (frequentist) meta-analysis [2, 3]. Although the numerical outputs of point estimates and intervals were not very different, they have different interpretations. More importantly, the measure used for hypothesis testing is distinct and will be further discussed.

In the example that used TAT for reducing anastomotic leaks, the Bayesian estimates were more conservative, and the intervals were slightly narrower. The 95% CrI keeps getting narrower as more studies are added, and this is better evident in the second example using six RCTs on the use of ICG in the cumulative meta-analysis forest plot. The frequentist CI from a single study provides very little information on what the true estimate would lie, since by principle, frequentist methods rely on repeated measurements. In contrast, the Bayesian CrI directly provides the interval that would contain the true estimate. Often the CI are thought to offer the same inference as CrI; however, this is a common but incorrect interpretation.

The next fundamental difference is the method used for hypothesis testing. The frequentist *p*-values were 0.15 for the use of TAT. Another common misunderstanding is that there was a 15% probability of H_0_ and 85% for H_1_ with the provided p-value. Again, this is incorrect, and, as explained in the preceding section, a *p*-value only means that the probability of observing the given data is 15% provided the H_0_ is true. No indication about the hypothesis being tested (H_1_) is offered by the frequentist output. Even if the H_0_ was less probable, a *p*-value cannot propose any insight into our H_1_ (use of TAT) unless one assumes that H_1_ is a complement of H_0_ [P(H_1_) = 1 – P(H_0_)]. However, this is not true since frequentists rely on long-run frequencies and do not assign direct probabilities to H_0_ or H_1_. The BF_10_, on the other hand, gives exactly what one would want: the ratio of probabilities of observing the data given that H_1_ was true to that if H_0_ was true. Thus, for the TAT example, a BF_10_ of 0.68 tells us that the null is 1.46 times more likely as compared to H_1_, given the data. The BF_10_ is much more informative and non-restrictive compared to the *p*-value, where an orthodox significance level is used to reject or accept the H_0_.

In the other meta-analysis of ICG use, the frequentist output was significant at traditional thresholds. A *p*-value of 0.0005 suggests that the probability of observing such data if the H_0_ was true is exceedingly low. However, only the BF_10_ gives the required information on how likely the H_1_ (ICG reducing anastomotic leaks) is over the null with the observed data. The BF_10_ indicated that it was nearly 20 times more likely to obtain the results under the condition that H_1_ was true. Although the BF_10_ estimate appears to be much more conservative than the *p*-value in this instance, the essence is to remember that the *p*-value provides no information about the H_1_, a point that needs reiteration. A special case may be considered wherein H_1_ is assumed to be a complement of H_0_, that is, ICG was the only other reason for the reduction in anastomotic leaks, the frequentist estimation of the likelihood ratio would be 199 [LR = (1-p)/p], an extreme overestimation by approximately tenfold (BF_10_ = 19.6) despite assuming non-informative priors. Thus, assuming frequentist calculations as a special case of Bayesian is also flawed.

The other advantage of Bayesian methods is the possibility of performing sequential updating of evidence with new studies being added without the need for correcting for multiple hypothesis testing [15]. The cumulative method allows the use of previous studies as a prior for forthcoming studies to be tested. Thus, similar to how everyday heuristics work, the prior beliefs are updated by new evidence rather than disregarding previous data. In the cumulative Bayesian meta-analysis forest plot for the ICG review, every study added chronologically does not seem to change the point estimates considerably; however, the CrI becomes more precise (Fig. 3). Similarly, the trend of BF_10_ is seen in the sequential BF_10_ plot (Fig. 2). As more studies are added that provide estimates in the same direction as others, irrespective of the magnitude, the BF_10_ keeps increasing. In other words, the likelihood or confidence in the H_1_ increases. The threshold of what should be considered significant is left for the end-users to decide; this gives much more freedom for the interpretation and application of the data rather than being rigidly categorical around a certain number (*p*-values < 0.05).

Several other modifications can be made within the Bayesian meta-analytic framework that the present deliberation has not addressed. Researchers familiar with meta-analysis will recognize that a FE or RE assumption needs to be made a priori. The present study used Bayesian model–averaged methods that do not make any such assumptions, assume a similar probability for each of these models, and average the result over them. Moreover, a BF_rf_ is also a usual part of the output that conveys the probability of observing the data under the RE over the FE hypothesis. A necessary aspect of Bayesian statistics is the prior distribution. Although the assumption of the prior may appear subjective, one can always model the outcome based on multiple priors (and distribution of priors), commonly using the Markov chain Monte Carlo methods. For the most conservative outputs, one can begin with a non-informative prior, as used in the examples cited, and this is one reason why the numerical summary estimates of the frequentist and Bayesian outcomes were similar. Had the experimentation used informative priors, the outcomes could have been different to some degree.

In Bayesian terms, the updated belief (posterior odds) is the numerical product of the prior odds and the BF. Now, if the prior odds become large enough, it would require even larger negative evidence to overcome the influence of the prior. If the prior becomes strong due to accumulating evidence, it will increasingly dominate the analysis. This means new trials will have less influence on shifting the posterior distribution unless they provide substantially contradictory evidence. In such cases, one may decide to avoid additional studies on the topic, similar to adaptive designs in Bayesian randomised trials, without the risk of alpha spending [16].

### Limitations

The discussion intentionally did not cover many other aspects of the Bayesian meta-analysis. The manuscript aimed to highlight the interpretative differences between Bayesian and frequentist meta-analyses rather than providing a comprehensive overview of Bayesian methods. Next, the study employed non-informative priors, specific prior distributions, and model-averaged methods based on prior subject knowledge. These choices were made to provide conservative estimates and emphasize the differences between Bayesian and frequentist approaches. However, this also means that other possible variations and modifications within the Bayesian framework were not explored. The manuscript deliberately avoided the use of formulae and mathematical discourse to keep the focus on the key interpretative differences. While this makes the content more accessible, it might limit the depth of understanding for readers who are seeking detailed methodological insights.

Although the manuscript used null priors to demonstrate conservative estimates, it acknowledges that the selection of priors in Bayesian analysis can be subjective. The outcomes could differ if informative priors were used, which were not explored in this study. The cumulative Bayesian methods can be particularly sensitive to the sequence in which studies are added, and thus, their use should be restricted to situations when a defined chronological pattern is evident. The discussion was based on specific examples using TAT and ICG in colorectal anastomosis. The conclusions drawn might not be fully generalizable to other contexts or types of studies. Finally, any of the meta-analytic methods apply to comparable studies only. If clinical heterogeneity is deemed significant, no complex computations can overcome the methodological differences enough to allow synthesis.

Every method of analysis has its application in the correct context. If a summary measure alone is required and the trials are relatively homogeneous, a frequentist meta-analysis is a good option. If there is prior knowledge that should be incorporated, a Bayesian meta-analysis is a strong choice, especially if the direct probability of the hypothesis being tested is needed. If trials are sequential or new trials have to be modelled into existing analysis, a cumulative Bayesian analysis with updating is ideal. As Pierre-Simon Laplace famously remarked: “Probability theory is nothing but common sense reduced to calculation” one should not restrict oneself to frequentist approaches alone when the question demands a conditional probability.

## Conclusion

While frequentist and Bayesian meta-analyses may produce similar point estimates based on prior evidence, their interpretations and implications for hypothesis testing differ significantly. Bayesian methods offer a more flexible, intuitive, and informative approach, particularly in contexts with prior knowledge or when sequential updating of new trials is required. While frequentist outputs depend on multiple experiments, assuming that the null is true, and heavy dependence on conventional *p*-value thresholds, Bayesian outputs provide the direct probability of the hypothesis in question and credible intervals that are likely to contain the true estimate.

## Data Availability

No new data was generated. All data were from previously published and referenced meta-analyses.

## Abbreviations

*BF*_10_: Bayes factor
*CI*: Confidence intervals
*CrI*: Credible intervals
*FE*: Fixed effects
*H*_0_: Null hypothesis
*H*_1_: Alternate hypothesis
*ICG*: Indocyanine green
*OR*: Odds ratio
*RCT*: Randomized controlled trials
*RE*: Random effects
*TAT*: Trans-anastomotic tube
